# Decreasing Patient Visit Length at a Student-Run Free Clinic via a Continuous Quality Improvement Project

**DOI:** 10.7759/cureus.66511

**Published:** 2024-08-09

**Authors:** Miranda J Reid, Ethan Kramer, Alexandria Iakovidis, Jamie B Harris, Rene M Kronlage, Amy Stanley, Carolyn Holland

**Affiliations:** 1 Department of Health Outcomes and Biomedical Informatics, University of Florida College of Medicine, Gainesville, USA; 2 Department of Emergency Medicine, University of Florida College of Medicine, Gainesville, USA

**Keywords:** patient's satisfaction, continuous quality improvement (cqi), plan-do-study-act (pdsa), quality improvement research, student-run free clinic

## Abstract

Introduction: The University of Florida Equal Access Clinic Network (EACN) is the largest student-run free clinic (SRFC) network in Florida. This student-driven, continuous quality improvement (CQI) project is intended to decrease total patient visit length at Eastside clinic, one of EACN’s primary care sites. The original median visit length of 126.25 minutes represented a significant time burden for patients, especially those with limited transportation or inflexible schedules.

Methods: Over six months, four Plan-Do-Study-Act (PDSA) cycles were implemented. PDSA cycle 1 increased personnel and space for taking vitals. PDSA cycle 2 reduced redundancy in the intake process. PDSA cycle 3 triaged patients to match patient complexity with student experience level. PDSA cycle 4 introduced “nudge” interventions to reinforce clinic flow. Total patient visit length and time spent at each step of clinic flow were recorded anonymously for each patient visit. The median visit length per week was tracked on a run chart.

Results: From PDSA cycle 1 through PDSA cycle 4, the median visit length decreased from 126 minutes to 114 minutes. This shift was primarily driven by a decrease in the length of patient intake from a median of 19 minutes to 9 minutes. The run chart did not show clear trends until PDSA cycle 4, which demonstrated a strong downward trend.

Conclusion: This study demonstrated the ability of a student-driven CQI model to decrease patient visit length in an SRFC setting. Similar models could be used to address this and other contributors to patient experience across SRFCs nationwide.

## Introduction

Student-run free clinics (SRFCs) provide high-quality care to marginalized populations, including the uninsured, underinsured, and undocumented [1]. A survey conducted in 2014 characterized the rapidly growing presence of SRFCs, identifying SRFCs at 106 of the 141 US Association of American Medical Colleges member institutions, with the majority of medical students at these institutions participating [2]. In addition to improving access to care, SRFCs have been shown to improve outcomes in a variety of conditions and specialties [3]. Multiple satisfaction surveys indicate patients are satisfied with the majority of the aspects of their clinic experience [4-7]. Evidence also suggests patients who attend SRFCs are less likely to make emergency department visits for non-urgent concerns, reducing the burden on local healthcare systems [3,8-13]. Finally, SRFCs are an effective educational environment for medical students to learn about social determinants of health, healthcare management, and interdisciplinary care [1,14,15].

While SRFCs have the capacity to positively impact patients, students, and the communities in which they operate, surveys of patient experience at SRFCs reveal areas for improvement [4-7]. Among published patient satisfaction surveys at SRFCs, the most commonly reported metric with the lowest patient satisfaction was waiting time [4-7]. Given the strong inverse relationship between patient satisfaction and waiting times, SRFCs have much to gain from making improvements in this area [16]. Despite this problem being well reported, few studies at SRFCs have described successful quality improvement solutions to decrease patient waiting times [17]. Furthermore, there are no published quality improvement projects at SRFCs aimed at decreasing waiting times using evidence-based, continuous quality improvement (CQI) methods. 

CQI varies in implementation; however, there are three common features: (1) systematic data-guided activities, (2) iterative testing and development, and (3) designs made with local conditions in mind [18]. During the planning process, opportunities for improvement of total patient visit length were identified through process maps and a key driver diagram developed through discussion by the authors. Process maps depict the sequence of events in a system from all relevant perspectives [19]. The key driver diagram identifies ideas for change and how these ideas affect key leverage points in a system [19]. Once opportunities for improvement were identified, our CQI project was conducted using iterative Plan-Do-Study-Act (PDSA) cycles. PDSA cycles consist of four stages: (1) identifying an opportunity for change (“plan”), (2) testing the proposed change (“do”), (3) analyzing the effects of the change (“study”), and (4) proposing adaptations and next steps for a new cycle (“act”) [20]. By using a standardized format continuously informed by data, PDSA cycles minimize risks associated with change [20]. Additionally, this manuscript was developed using the Standards for Quality Improvement Reporting Excellence (SQUIRE 2.0), a commonly used reporting standard in CQI [21].

During the summer of 2022, the University of Florida College of Medicine Equal Access Clinic Network (EACN) launched a multi-stage CQI project with the aim of decreasing total patient visit length. EACN comprises a robust network of SRFCs, offering primary care services four nights per week, as well as “specialty nights” in gynecology, LGBTQ+ health, pediatrics, prenatal health, psychology, psychiatry, ophthalmology, physical therapy, occupational therapy, dermatology, and cardiology. The interdisciplinary care and high level of student, faculty, and community support position EACN as an ideal setting for running, analyzing, and modeling quality improvement projects. Here, we describe the experience and outcome of a quality improvement project with the aim of decreasing total patient visit length at the Eastside clinic, an EACN primary care site, from a median of 126 minutes to a median of 90 minutes over a six-month period to closer align it with the clinic time in a typical primary care visit [22]. This article was previously presented as a poster at the Institute for Healthcare Improvement (IHI) Forum on December 10-13, 2023.

## Materials and methods

Context

**Figure 1:**
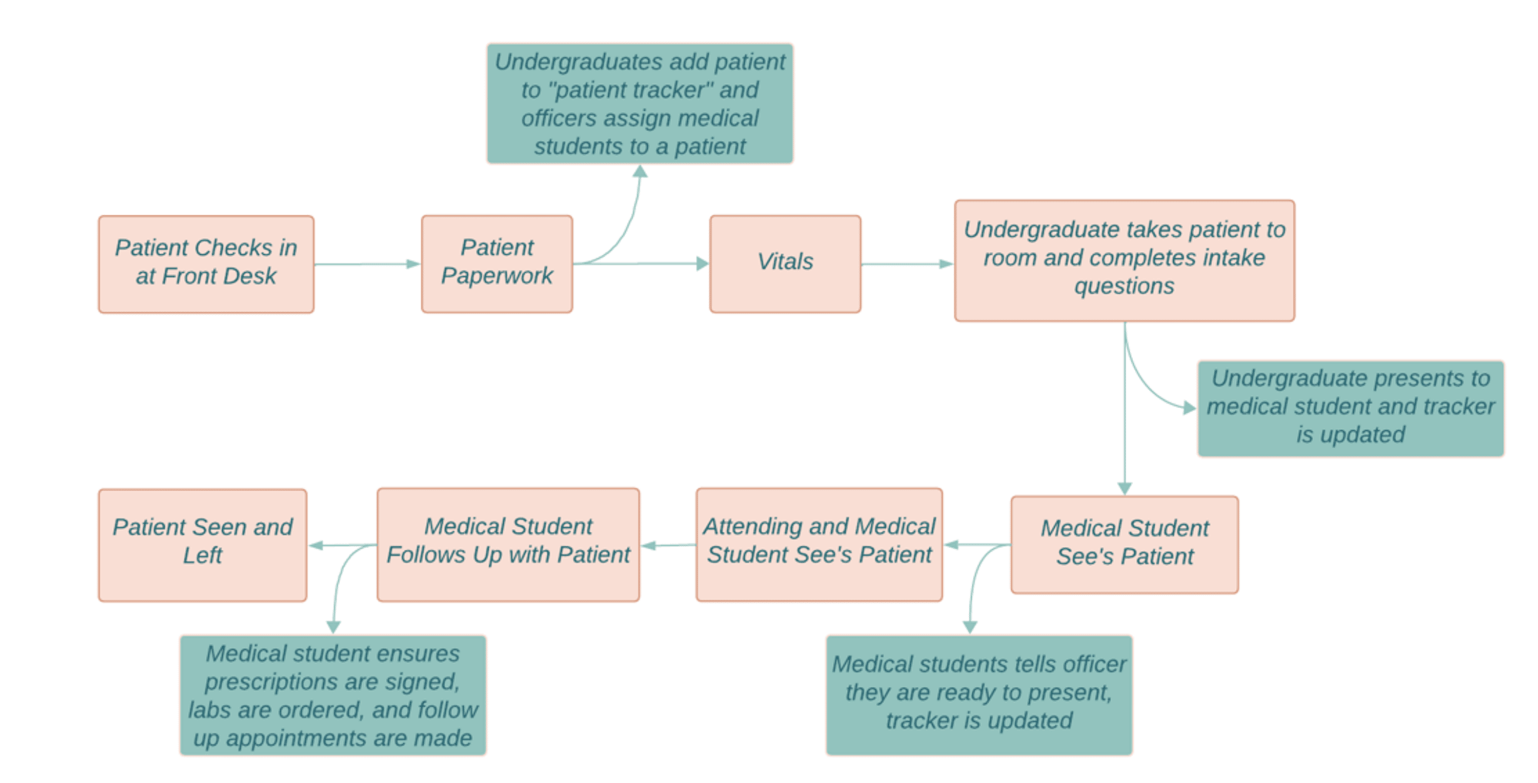
Eastside clinic flow prior to initiation of PDSA cycle 1. PDSA, Plan-Do-Study-Act Image Credit: Alexandria Iakovidis, Co-Author

The EACN Eastside clinic site primarily cares for uninsured and underinsured adult patients every Tuesday from 5:00 p.m. until 9:00 p.m. The clinic is primarily operated by medical students with oversight by attending physicians, resident physicians, and advanced practice providers (APPs). Additionally, undergraduate students assist with patient intake, student pharmacists assist with prescriptions and medication reconciliation, and graduate student psychologists offer on-site psychological services. Eastside clinic offers weekly primary care services as well as monthly dermatology and psychiatry services and biweekly LGBTQ-specific services. In 2022, Eastside clinic had over 600 visits. The CQI team included the medical student director of EACN, Eastside clinic medical student officers, and additional medical student volunteers. Clinic flow prior to PDSA cycle 1 is described in Figure 1.

In brief, patients were 1) checked in and roomed by undergraduate volunteers ("intake"), then 2) seen individually by a medical student volunteer ("student seeing"), before waiting in their room while the student presented the case to the physician or APP ("waiting"), and finally 4) seen jointly by the medical student and physician or APP ("MD seeing"). Preliminary baseline data for patient length of stay was collected from weeks one to eight. The aim of this project was to decrease the median total patient visit length per week from 126.25 minutes to 90 minutes over a period of six months.

PDSA cycles

One of the key drivers identified was a slow patient intake process. It was hypothesized that a central vital signs collection process would decrease patient intake time. PDSA cycle 1 ran from week nine to sixteen and streamlined the vital sign collection workflow by designating a centralized “vitals station” for the collection of patient vitals by Eastside medical student officers solely responsible for vital sign collection. This was a change from a decentralized model, where individual undergraduate student volunteers would collect vital signs during the process of rooming patients. Additionally, this change addressed a new institutional administrative policy prohibiting undergraduate volunteers from collecting patient vitals.

PDSA cycle 2 ran from weeks 17-19 and aimed to reduce redundancy in the intake process by introducing a standardized intake form including multiple elements of past medical history (chronic conditions, allergies, and current medications) and a review of systems. The previous form was less comprehensive and resulted in undergraduate volunteers repeating the history of illness with patients in the exam room. This created further redundancies, as the patients would then repeat their histories and review of systems again with both the medical student volunteers as well as the physicians/APPS. Adapting this intake form thus eliminated the need for undergraduates to take a verbal history of illness.

PDSA cycle 3 ran from weeks 20-23 and focused on triaging routine chief concerns, such as prescription refills, to first-year medical student volunteers and resident physicians while allocating more complex or acute chief concerns to 2nd-4th year medical student volunteers and attending physicians. The medical student clinic director in conjunction with the medical student officer in charge of assigning volunteers and physicians identified patients to be triaged. This change aimed to eliminate the need for attending physicians to directly participate in every patient interaction, which was causing the delay of the discharge for patients with simpler concerns. Prior to this intervention, resident physicians commonly consulted attending physicians regarding the management of complex concerns, often requiring the attending physician to step away from their own patient visits, prolonging multiple patient visits.

PDSA cycle 4 ran from weeks 24-31 and involved establishing “nudge” interventions to remind medical student officers and volunteers of clinic flow. This included displaying signs around the clinic at key transition locations to remind volunteers of the next steps in clinic flow, as well as how to receive administrative assistance with the next steps listed. For example, signs near the administrative office listed when, where, and how to obtain any necessary prescriptions and orders for medical testing.

Analysis

Time intervals, the number of physicians volunteering in the clinic, medical/PA student academic year, as well as the number of total patients, new patients, and specialty night patients were collected in an online, confidential, shared spreadsheet (“tracker”). This tracker was created and used prior to this project as a standardized way to organize and monitor clinic flow. No identifying patient information was entered into the tracker. The tracker was programmed to record the length of time spent on each individual step in clinic flow in addition to the total patient visit length. Total visit length was defined as the time from the patient initiating the sign-in process at the front desk to the time the patient left the clinic. After each week, the QI team downloaded de-identified data to preserve for later analysis.

Due to a non-parametric distribution of visit length as confirmed by the Shapiro-Wilk test (p<0.01), the median and interquartile range were calculated for each time interval for each PDSA cycle using R Statistical Software v4.2.2. With the non-parametric distribution and number of cycles, the Kruskal-Wallis test was the most appropriate to determine if any of the medians significantly differed between PDSA cycles. Medians, interquartile ranges, and Kruskal-Wallis tests were also used to assess if there were any differences in the number of patients, new patients, physicians, or student academic years across PDSA cycles. Student academic year was measured based on the proportion of patients from that week seen by a student of that year and their graduate school. Patients seen by multiple students working together were grouped into an additional category. To assess whether the number of total patients, new patients, specialty night patients, physicians, or the student academic year had an impact on visit length, the Pearson correlation coefficient "r" was calculated to assess the correlation between each of these continuous variables and the weekly median visit length. The median total patient visit length per week was plotted in a run chart for the length of the project period to assess for any shifts or trends.

Ethical approval for this quality improvement project was obtained from the executive director of EACN and the individual directors of each participating clinic. No identifying information was collected from patients or volunteers. The team did not access any patient medical records for this project. Finally, this project was registered with the UF Health Sebastian Ferrero Office of Clinical Quality and Patient Safety under project identification number 2295.

## Results

At the completion of each PDSA cycle, the Eastside clinic director decided to adapt, adopt, or abandon the intervention into the routine clinic standard practices. PDSA cycles 1, 2, and 4 were adopted as described. PDSA cycle 3 was abandoned due to the logistical difficulty associated with identifying the complexity of patient complaints. All interventions implemented in all four PDSA cycles were refined week to week based on stakeholder (director, officer, and volunteer) feedback for the duration of each PDSA cycle. The median number of total patients, new patients, and physicians remained similar across PDSA cycles (p<0.05) (Table 1).

**Table 1:** The median number of patients, new patients, and physicians present per night during a given PDSA cycle. The interquartile range is included in brackets. The Kruskal-Wallis test was used to assess for any significant variation in these characteristics between PDSA cycles and no statistically significant variation was found (all p>0.05). *Data on physicians present was not collected during the baseline period. PDSA, Plan-Do-Study-Act

PDSA cycle	0	1	2	3	4	χ^2^	df	p-test
Patients	9.00 (8.00, 10.25)	12.00 (10.50, 15.50)	12.50 (9.50, 16.50)	10.50 (7.75, 13.25)	14.00 (11.00, 15.50)	6.146	4	0.189
New patients	2.00 (1.00, 2.00)	2.00 (1.50, 3.50)	3.00 (2.75, 3.25)	0.50 (0.00, 1.75)	3.00 (2.75, 4.00)	6.883	4	0.142
Physicians	NA (NA, NA)*	3.00 (3.00, 3.00)	4.00 (3.75, 4.00)	3.00 (3.00, 3.00)	3.00 (3.00, 4.00)	4.901	3	0.179

There was a weak negative relationship between the number of physicians (r=-0.31) and the number of patients present for psychology night (r=-0.23) and the median weekly visit length. There was no relationship between the total number of patients (r=-0.07), the number of new patients (r=-0.04), or number of patients present for LGBTQ night (r=0.13) or dermatology night (r=-0.11), and the weekly median visit length. The proportion of patients seen by either an MS1, PA1, or multiple students varied significantly between cycles (p<0.05) (Table 2).

**Table 2:** The median proportion of patients seen by students in a given graduate school year. The interquartile range is included in brackets. The Kruskal-Wallis test was used to assess for any significant variation in proportion seen between PDSA cycles. The proportion of students seen by an MS1, PA1, and the proportion seen by multiple students varied significantly between cycles (p<0.05). *Statistical significance at a level of p<0.05. **Statistical significance at a level of p<0.01. PDSA, Plan-Do-Study-Act

Student year	0	1	2	3	4	χ^2^	df	p-test
MS1	0.00 (0.00, 0.00)	0.00 (0.00, 0.04)	0.05 (0.00, 0.10)	0.14 (0.07, 0.25)	0.27 (0.10, 0.42)	14.782	4	0.005**
MS2	0.39 (0.23, 0.69)	0.17 (0.07, 0.42)	0.52 (0.47, 0.55)	0.26 (0.20, 0.61)	0.21 (0.10, 0.41)	4.431	4	0.351
MS3	0.12 (0.08, 0.15)	0.07 (0.00, 0.24)	0.08 (0.06, 0.11)	0.20 (0.05, 0.21)	0.15 (0.09, 0.19)	1.464	4	0.833
MS4	0.05 (0.00, 0.11)	0.00 (0.00, 0.08)	0.06 (0.04, 0.08)	0.00 (0.00, 0.00)	0.00 (0.00, 0.00)	8.609	4	0.072
PA1	0.00 (0.00, 0.00)	0.00 (0.00, 0.00)	0.00 (0.00, 0.03)	0.00 (0.00, 0.00)	0.22 (0.10, 0.31)	17.092	4	0.002*
Multiple students	0.45 (0.08, 0.55)	0.30 (0.24, 0.68)	0.20 (0.19, 0.21)	0.14 (0.00, 0.34)	0.00 (0.00, 0.07)	11.965	4	0.018*

The proportion of patients seen by multiple students had a weak positive relationship with visit length (r=0.23), and the proportion seen by an MS3 had a weak negative relationship with visit length (r=-0.24). There was no relationship between the proportion of patients seen by an MS1 (r=-0.10), MS2 (r=0.03), MS4 (r=0.09), or PA1 (r=-0.09) and the weekly median visit length.

The median total visit length decreased by 10% from 126.25 minutes (before PDSA cycle 1) to 113.5 minutes (after PDSA cycle 4), which did not reach the original goal of decreasing the median visit length to 90 minutes. While there was a significant decrease (p<0.05) in the amount of time spent on intake across successive PDSA cycles, from a median of 19.00 minutes to 9.00 minutes by PDSA cycle 4, the overall amount of time patients spent face-to-face with physicians significantly increased (p<0.05) by the end of the project (Table 3).

**Table 3:** The median time for each step in the clinic flow per patient per night during a given PDSA cycle. The interquartile range is included in brackets. The Kruskal-Wallis test was used to assess for any significant variation in time intervals between PDSA cycles. Both the time for intake and the time the patient was being seen by a physician varied significantly between cycles (p<0.05). *Statistical significance at a level of p<0.05. PDSA, Plan-Do-Study-Act

PDSA cycle	0	1	2	3	4	χ^2^	df	p-test
Intake	19.00 (10.75, 22.75)	12.50 (11.75, 16.50)	13.00 (11.75, 13.2)	9.50 (8.00, 11.00)	9.00 (8.88, 10.50)	9.758	4	0.045*
Student seeing	20.75 (19.12, 31.00)	24.00 (22.75, 24.75)	28.00 (23.75, 33.75)	20.75 (19.12, 32.50)	21.75 (19.88, 25.00)	2.544	4	0.637
Waiting	13.25 (7.38, 21.00)	7.00 (6.25, 14.75)	11.25 (5.00, 17.62)	11.75 (9.38, 16.75)	7.50 (6.38, 14.25)	1.561	4	0.816
MD seeing	36.75 (34.25, 47.25)	28.50 (27.00, 35.50)	30.50 (25.25, 34.0)	43.50 (38.88, 44.38)	40.50 (33.88, 48.50)	10.058	4	0.039*
Total time	126.25 (112.00, 128.75)	116.00 (106.00, 123.00)	123.50 (114.50, 125.12)	122.75 (116.62, 130.62)	113.50 (109.50, 131.88)	2.0455	4	0.727

**Figure 2:**
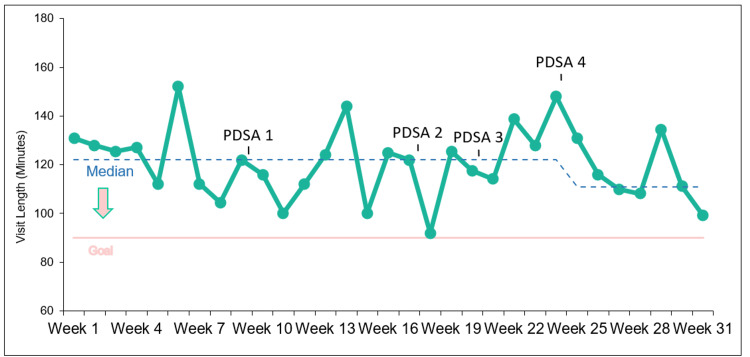
Run chart of median length of stay per patient per night. PDSA, Plan-Do-Study-Act

This is reflected in the run chart (Figure 2), which does not show any clear trends in the median length of stay per week until PDSA cycle 4, where there is a strong downward trend in total patient visit length.

This trend is only broken by one data point, which corresponds to the clinic held on February 14, 2023. Likely due to Valentine’s Day, there was a decrease in both student volunteers and administrators for the clinic, which coincided with an unexpectedly high patient volume of 18 (compared to the median of 14 (IQR: 11-15.5) for PDSA cycle 4). Weeks (n=2) during which data for visit length was missing for more than 50% of patients were excluded from the run chart and overall analysis.

## Discussion

Using CQI methods, this project achieved a 10% reduction in median total patient visit length by PDSA cycle 4. Given this was the first CQI project implemented at the Eastside clinic, our initial goal to decrease visit length by greater than 25% was potentially too ambitious. The 10% reduction was accomplished via a reduction of both intake time and time patients spent waiting to be seen by a licensed provider after being seen by a medical student (Table 3). The overall decrease in visit length was associated with an increase in the amount of time spent on face-to-face patient care; notably, there was a statistically significant increase in the amount of time patients spent with physicians/APPs (Table 3). This suggests that not only were visit lengths shorter, but patients spent more time than before actively receiving care, improving the overall patient experience. Additionally, this project achieved these results while focusing on small tests of change that should be both feasible and acceptable in other SRFC settings. SRFCs by nature have inherent limitations on time and financial resources, making it difficult to adapt interventions utilized in other clinic or hospital settings. Our project highlights the use of straightforward, free strategies to reduce total patient visit length.

While the median total visit length was lower in each individual PDSA cycle than in the pre-implementation period (median=126.25), the difference between baseline data and PDSA cycles 2 (median=123.50) and 3 (median=122.75) were fairly minor. PDSA cycle 1 showed a more meaningful decrease in patient visit length (median=116.00), but unlike PDSA 4 (median=113.50), there were no clear trends or signals in the run chart for PDSA cycle 1 (Figure 2). PDSA cycles 1 and 2 focused on refining different portions of the intake process and both showed a decrease in the length of intake time compared to baseline (Table 3). These cycles’ changes were adopted and may have contributed to continued decreased intake times throughout the rest of the project period. However, the variation in time spent on other portions of the visit limited the overall impact of these cycles. PDSA cycle 3 was not adopted due to the logistical challenges of properly triaging the complexity of patient visits. Due to this challenge, the cycle was applied to a relatively limited number of patients each week, limiting its utility and impact on time. PDSA cycle 4 showed the strongest trend of any cycle with a steady decrease, except for the clinic held on Valentine’s Day. This cycle may have been more successful as the “nudge” intervention had the potential to impact multiple stages of the visit, leading to both faster intake and decreased amount of time patients spent waiting to be seen by a physician/APP.

PDSA cycles 1-3 occurred earlier in the academic year, which may have added additional challenges to decreasing visit length. The start of pre-implementation data collection coincided with the start of new PA students volunteering in the clinic, while the start of PDSA cycle 1 coincided with the beginning of a new medical school year. This meant first-year medical and PA students were volunteering at EACN for the first time, and often shadowed more experienced students or saw patients in pairs. This led to a statistically significant increase in the proportion of patients seen by multiple students (Table 2), which steadily decreased from the pre-implementation period (45% of patients) to PDSA 4 (0% of patients). The proportion of patients seen by multiple students had a weak positive relationship with visit length (r=0.23), as older students spent time acquainting first-year students with clinic flow, history taking, and the electronic medical record. This may have contributed to the increased visit length during these cycles. However, if this was a stronger contributor to visit length, PDSA cycle 1 would have been expected to have a considerably higher visit length. Other contextual factors, like the number of physicians, total patients, or new patients, did not vary significantly between cycles (Table 1).

There are few existing studies addressing patient visit length in the SRFC setting and none using CQI methodology to address it [17,23,24]. However, the limited evidence does suggest that strategies used in PDSA cycle 4, which resulted in the clearest improvement in our setting, have worked in other SRFCs. For example, Lee et al. also focused on improving communication about what stage in the visit patients were in to decrease the amount of time patients were not being seen by either students or providers and impact [17]. Lee et al. rolled out 17 total interventions at the same time resulting in a shorter patient visit while preserving the length of time patients and providers spent actively engaging, as was seen in our study. However, as all interventions were concurrent, they were not able to isolate which contributed to the improved visit flow. Several of the interventions tried in this setting did not result in any improvement in our study, like streamlining the measurement of vital signs. The sequential rollout of interventions in our study could allow future SRFCs addressing visit length to focus on a narrower set of possible interventions that have demonstrated success. This includes strategies that have been proposed by others but not previously tested. Bu et al. suggested that a complexity-based triage system could help minimize wait times [25]. However, our experience in PDSA cycle 3 showed that any time saved by assigning a complex patient to providers with the appropriate training level was offset by time spent waiting for a provider of the appropriate level to become available. Additionally, the small number of highly complex patients in our SRFC setting made it difficult for any changes to impact overall patient visit length, indicating that this strategy may have more utility if implemented in a setting with higher overall patient volume, more providers, or a higher proportion of complex patients. Some strategies shown to be successful in the literature, like the introduction of an online scheduling tool or limitations on the time allowed for students to collect patient history, were unable to be evaluated in our study as they were already a part of the existing clinic flow [23,24]. However, anecdotal evidence from student volunteers and administrators suggests that these are seen as valuable baseline strategies and are believed to contribute to patient satisfaction and clinic efficiency.

We identified several limitations of this project that could be addressed in future studies. The most significant limitation of this project was the lack of precision in measurement. A pre-existing online tracker was used in this project to increase the feasibility and acceptability of data collection and minimize disruption to workflow during the project period. This meant the medical student volunteers collecting times were not project personnel, leading to times being updated up to several minutes after patient status had changed, as well as occasional lapses in data collection. This limitation was present in all PDSA cycles and, therefore, should not impact overall trends in the data. Additionally, weeks for which more than 50% of data was missing (n=2) were excluded from the analysis. Using pre-existing tracking tools (e.g., times in an EHR) is common practice in CQI studies. However, future CQI projects should consider whether collecting time via direct observation would meaningfully increase the internal validity of the project. The pre-existing tracking tool for this project did not allow for an independent assessment of patient complaint complexity, which given the small number of patients per week could have meaningfully impacted visit length. Another limitation was concluding data collection with the end of PDSA 4, limiting the ability to assess the long-term impacts of the intervention and intervention sustainability. Future projects could continue collecting data beyond the active intervention period to address these concerns. Finally, we did not formally measure patient satisfaction and instead relied on decreasing times to improve the patient experience. Future studies could include patient satisfaction as a balancing measure to ensure that the patient experience is maintained or improved even as the clinic flow changes.

## Conclusions

This project demonstrated the potential for student-led CQIs to address patient visit length and more generally improve the patient experience at SRFCs. Due to time and financial constraints in this setting, designing interventions that are feasible and continuously adapting them with stakeholder feedback to ensure acceptability is essential. In this project, the most successful strategy reduced time at multiple stages across the visit. Similar strategies could be utilized at SRFCs across the country. These projects have the added benefit of also serving as an essential training opportunity to improve medical students’ knowledge and experience about CQI methods. Future studies could more directly measure patient satisfaction, ensuring that the decrease in visit length leads to a meaningful improvement in patients’ experiences.
